# Detection of Defective Lettuce Seedlings Grown in an Indoor Environment under Different Lighting Conditions Using Deep Learning Algorithms

**DOI:** 10.3390/s23135790

**Published:** 2023-06-21

**Authors:** Munirah Hayati Hamidon, Tofael Ahamed

**Affiliations:** 1 Graduate School of Science and Technology, University of Tsukuba, 1-1-1 Tennodai, Tsukuba 305-8577, Japan; hamidon.munirah.ws@alumni.tsukuba.ac.jp; 2 Institute of Life and Environmental Sciences, University of Tsukuba, 1-1-1 Tennodai, Tsukuba 305-8577, Japan

**Keywords:** seedling detection, lettuce, indoor farming, deep learning, YOLO, CenterNet, faster RCNN

## Abstract

Sorting seedlings is laborious and requires attention to identify damage. Separating healthy seedlings from damaged or defective seedlings is a critical task in indoor farming systems. However, sorting seedlings manually can be challenging and time-consuming, particularly under complex lighting conditions. Different indoor lighting conditions can affect the visual appearance of the seedlings, making it difficult for human operators to accurately identify and sort the seedlings consistently. Therefore, the objective of this study was to develop a defective-lettuce-seedling-detection system under different indoor cultivation lighting systems using deep learning algorithms to automate the seedling sorting process. The seedling images were captured under different indoor lighting conditions, including white, blue, and red. The detection approach utilized and compared several deep learning algorithms, specifically CenterNet, YOLOv5, YOLOv7, and faster R-CNN to detect defective seedlings in indoor farming environments. The results demonstrated that the mean average precision (mAP) of YOLOv7 (97.2%) was the highest and could accurately detect defective lettuce seedlings compared to CenterNet (82.8%), YOLOv5 (96.5%), and faster R-CNN (88.6%). In terms of detection under different light variables, YOLOv7 also showed the highest detection rate under white and red/blue/white lighting. Overall, the detection of defective lettuce seedlings by YOLOv7 shows great potential for introducing automated seedling-sorting systems and classification under actual indoor farming conditions. Defective-seedling-detection can improve the efficiency of seedling-management operations in indoor farming.

## 1. Introduction

Indoor farming has been increasingly popular as a method for producing crops such as leafy vegetables and fruits in a controlled environment [1]. Artificial light is regarded as the primary energy source required for photosynthesis as well as other physiological processes in crop production for indoor farming systems [2]. In this regard, seedling growth is a very important initial stage to ensure the quality of products during harvesting.

Proper seedling management on indoor farms is a key to ensure healthy and vigorous plants that produce high yields [3]. One of the factors that can affect seed germination is the emergence rate, which refers to the percentage of seeds that successfully grow into seedlings. Despite optimal germination conditions, some seeds may fail to germinate due to a range of factors, such as genetic variability, seed damage, environmental influences, or inappropriate storage conditions. However, even if all the seeds germinate, there can be discrepancies in the growth and development of individual seedlings [4]. There is also a possibility that multiple seedlings grow in a single seedling hole. This can happen accidentally during the sowing process and is more likely to occur with very light and tiny seeds such as lettuce [5]. Due to the small size of the seeds and the difficulty in handling them, it can lead to multiple seeds accidentally being placed in the same hole. This can cause uneven seed distribution, resulting in empty holes or seed clusters. The unintentional growth of multiple seedlings in a single seedling hole can lead to competition for resources, especially in indoor farming operations that rely highly on energy input for plant growth.

Hence, early identification and removal of poor-quality or defective seedlings are crucial in preventing significant losses in indoor farming. Currently, defective seedlings are sorted manually. It is a very laborious and time-consuming operation that can not only increase production costs but also reduce the overall efficiency of seedling-management operations [6,7]. Moreover, the manual sorting process in an indoor farm may need to be performed under complex light conditions, such as under varying LEDs spectra and intensities [3]. LEDs have become a prime option for indoor farming due to their advantages in providing precise and customizable lighting conditions. With LEDs, it is possible to design specific spectral combinations by selecting and combining different wavelengths of light. This flexibility allows growers to optimize light conditions for different crops and various growth stages of crops to achieve the highest yield [8].

For instance, blue light (wavelengths around 400–500 nm) is known to be crucial for promoting chlorophyll production, stomatal opening, and photosynthesis [9], while red light (wavelengths around 600–700 nm) can increase biomass accumulation, stem elongation, and leaf expansion [9,10]. By adjusting the ratios and intensities of these wavelengths as well as potentially incorporating other wavelengths, growers can optimize light conditions to achieve the highest yields and desired plant characteristics for specific crops and growth stages [11,12]. However, these complex lighting conditions of indoor farms can affect the visual appearance of seedlings [9], making it more challenging for employees to consistently identify and separate defective seedlings during the sorting process. Additionally, prolonged exposure to the visually uncomfortable lighting conditions created by these LED lights can cause eyestrain [13]. These challenges can lead to errors and inconsistencies, potentially resulting in reduced crop yields or quality issues. Thus, there is a need to implement an automated detection system to identify defective seedlings in indoor farms under different light conditions to improve efficiency and cost effectiveness while reducing the possibility of errors, high subjectivity, and low efficiency in manual sorting.

Some of the techniques being explored include using machine learning algorithms to analyze seedling images and detect signs of stress or abnormal growth patterns [14,15]. However, despite the progress made, there are still research gaps such as sensitivity to noise, low robustness, and limited adaptability to various environmental conditions, especially in indoor farms. These limitations need to be addressed for more effective and accurate detection of defective seedlings under indoor lighting conditions.

In recent years, computer vision and artificial intelligence (AI) have shown great potential for use in agriculture, especially deep learning (DL) based on convolutional neural networks (CNNs). CNNs have been widely used for various image-related tasks, such as image classification, object detection, and segmentation. In agriculture, CNNs are frequently used to analyze plant images for several purposes, including the detection of plant species [16,17], fruit counting [18,19,20], weed identification [21,22], and stress or disease detection [23,24,25,26]. Deep CNNs have also shown great potential in indoor farming environments [5,27,28]. However, the existing research has mainly focused on evaluating the detection performance of different CNN models only. There is a strong need for a comprehensive evaluation that not only compares the performance of different models but also assesses how well they perform in a range of indoor farming environments.

Therefore, the objective of this study was to detect defective lettuce seedlings in an indoor environment using image datasets collected under various indoor lighting conditions by using several state-of-the-art (SOTA) deep learning models. The comparative assessment outcomes from this study have the potential to contribute valuable insights into the feasibility and effectiveness of deep learning methods for seedling detection in diverse indoor farm environments to enable automated and efficient sorting systems in seedling management.

## 2. Related Works

Generally, two types of CNN architectures have been widely used in object detection, namely two-stage detectors and single-stage detectors. Two-stage detectors, also known as region-based detectors, typically involve two stages of computation. In the first stage, the model generates a set of candidate regions, known as region proposals, that are likely to contain objects. In the second stage, the model classifies the region proposals and refines their bounding boxes to localize the objects more accurately within them [29]. Thus, two- stage detectors typically have high localization and recognition accuracy. Examples of two-stage detectors include scalable sequential pyramid networks (SPP-Net) [30], faster R-CNN [31], and mask R-CNN [32]. One-stage detectors, in contrast, perform object detection in a single stage. These models use a CNN architecture to extract features from the input image and then apply several convolutional layers to predict object-bounding boxes and class probabilities. As they involve less computation, they boast high inference speed [26]. Examples of one-stage detectors include CenterNet [33], single-shot multibox detector (SSD) [34], and you only look once (YOLO) [35].

Both of these types of object detection have been utilized for seedling detection. For instance, Quan et al. introduced a maize-seedling-detection model using Faster R–CNN with VGG-19, which achieved a precision of 97.71% in detection under sunny, cloudy, and rainy conditions [36]. Oh et al. proposed a framework for cotton seedling detection and counting from high-resolution UAS imagery using the YOLOv3 object-detection model [37]. Li et al. developed an improved object-detection model based on faster R-CNN for high-precision detection of hydroponic lettuce seedlings in a greenhouse and achieved a mean average precision of 86.2% [5]. Fang et al. proposed a ginger seedling recognition network based on YOLOv4-LITE by replacing the original CSPDarknet53 backbone network with MobileNetv2 [38]. Liu et al. introduced a lightweight maize weed detection model using YOLOv4-tiny CNN combined with an attention mechanism and an improved SPP structure. The model can differentiate between maize seedlings with weeds such as Abutilon, Chenopodium, morning glory, and amaranth [39]. Zhang and Li developed ESPA based on the YOLOv5 attention mechanism to determine the survival rate of canola seedlings at different growth stages. The network achieved an average accuracy of 99.6% on a dataset containing 3368 images [27]. Tan et al. proposed a cotton-seedling-detection and -counting model using YOLOv4 combined with the optical flow method. The trained YOLOv4 model detected cotton seedlings with high accuracy under outdoor conditions of high occlusions, blurred pictures, complicated backgrounds, and extreme illumination [40].

While several studies have shown the potential of deep learning methods in detecting seedlings in field crop cultivation [36,38,39,40], there is a lack of research specifically focused on detecting seedlings in indoor farming environments, particularly those with varying lighting conditions. Although there are also few studies on detecting seedlings in indoor farming environments, most of them have typically been set up in uniform lighting conditions and have not explored the impact of lighting backgrounds on seedling detection [5,27,28]. In the case of indoor farming, lighting conditions can vary greatly depending on factors such as the type of light source used, the distance of the plants from the light source, and the positioning of the plants within the growing area. These conditions can affect the appearance of the seedlings, making them appear differently than they would under natural lighting conditions [2,3,9]. Given these limitations, it is important to carry out experiments specifically tailored to the conditions of indoor farming with different light settings to assess the performance of deep learning methods in seedling detection. Hence, this study proposes the detection of defective lettuce seedlings in an indoor farming environment using several SOTA deep learning algorithms.

## 3. Materials and Methods

**Figure 1:**
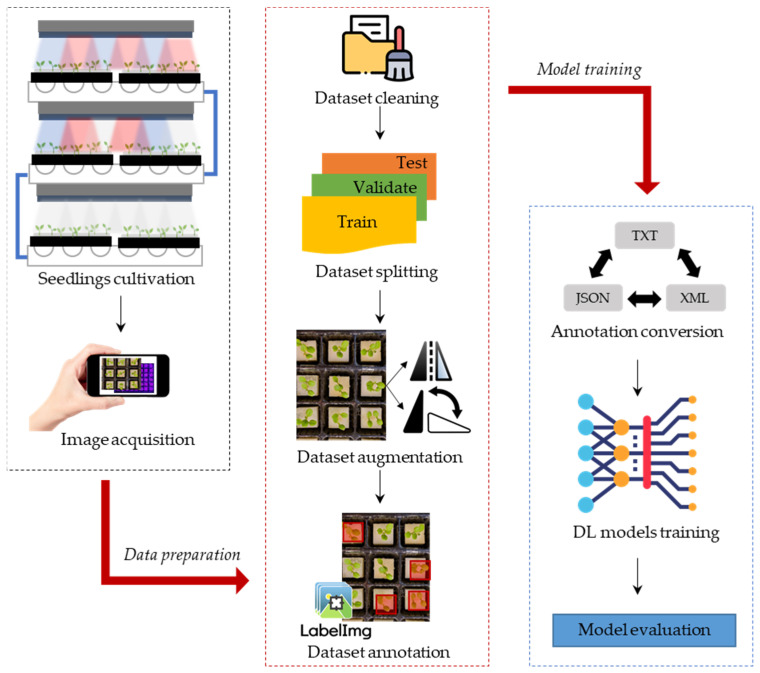
Seedling image acquisition process and development of the detection system.

This study started with a seed germination and seedling growth process for image acquisition to prepare the datasets for detecting defective lettuce seedlings. Then, a series of data-curation procedures were performed before training the DL models. These procedures involved various steps such as data cleaning to remove low-quality images, data splitting, data augmentation, and format conversion (Figure 1).

### 3.1. Plant Material and Cultivation Conditions

**Figure 2:**
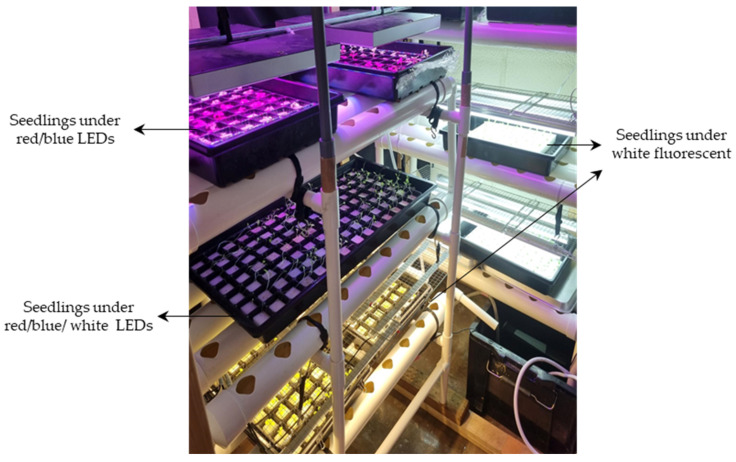
Lettuce seedling growth system under different indoor lighting settings.

Sunny green lettuce (Sakata Seed Corporation, Yokohama, Japan) was chosen as the experimental plant in this study. The experiment was conducted in a small-scale cultivation room located in the Bioproduction and Machinery Laboratory, Tsukuba-Plant Innovation Research Center (T-PIRC), University of Tsukuba, Japan. The lettuce seeds were sown in hydroponic seedling sponges, placed in seedling trays, and soaked with water. Each tray consisted of 48 holes. As there was no availability of public datasets for defective lettuce seedlings grown indoors, it became essential to generate a new dataset for the defective seedlings to facilitate the training of deep learning models. Half of the trays were intentionally sown with more than one seed per sponge to obtain the necessary defective seedling image datasets. All the trays were then arranged on a hydroponic cultivation system, with each level having different lighting conditions: a combination of red and blue LEDs; a combination of red, blue, and white LEDs; and white fluorescent (Figure 2). The seedling growth cycle was repeated three times.

### 3.2. Acquisition of Lettuce Seedling Images

**Figure 3:**
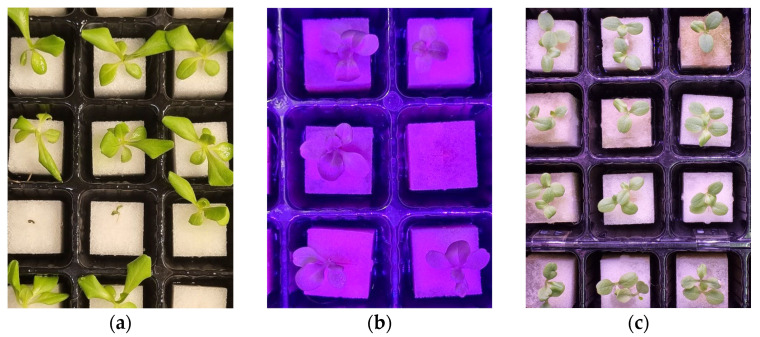
Example of captured images of lettuce seedlings under (**a**) white fluorescent; (**b**) red/blue LEDs; and (**c**) red/blue/white LEDs.

The seedling images were captured every other day for 15 days in each growth cycle, starting after the emergence of the first true leaves. A smartphone camera (Samsung Galaxy S21FE, Samsung Electronics Co., Ltd., Suwon, Korea) was used to obtain the image data from the top angle with the different distances and light conditions as mentioned in Section 3.1 (Figure 3). The resolution of each image was 3000 × 4000 pixels. Initially, the total dataset was 1124 images. However, after carefully examining and eliminating poor-quality, duplicate images or images unsuitable for annotation, 825 images were found suitable for further training on deep learning algorithms. Further, the image dataset was split into training, validation, and testing. The images were uniformly resized to 800 × 600 pixels to reduce the computational complexity, allowing for faster training times and more efficient resource utilization.

### 3.3. Dataset Preparations

#### 3.3.1. Data Augmentation

**Figure 4:**
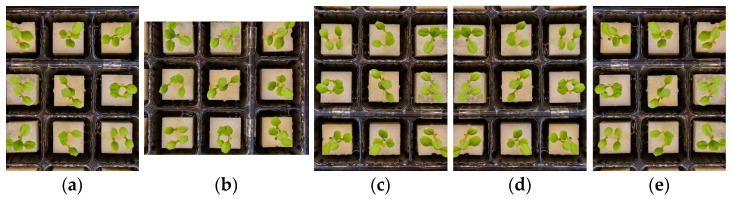
Different image augmentation methods: (**a**) original image; (**b**) 90° clockwise rotation; (**c**) 180° clockwise rotation; (**d**) horizontal flip; and (**e**) vertical flip.

Deep learning algorithms require a large dataset to accurately learn and extract features from images. However, collecting a vast dataset can be a time-consuming and challenging task. To overcome this challenge, data-augmentation techniques were employed randomly on the training dataset to increase the size and variability of the original training dataset. The data-augmentation techniques used in this experiment included rotation (clockwise 90 and 180 degrees) and flipping (horizontally and vertically) (Figure 4). As a result of these augmentations, the final training images numbered 2040.

#### 3.3.2. Data Annotation

Accurately specifying the location of defective seedlings in each image is important for an effective training process. An open-source software, LabelImg (https://github.com/heartexlabs/labelImg, accessed on 17 May 2023) was utilized to annotate the defective seedlings. In this study, the defective seedlings included non-germinated seeds, dead seeds, and multiple seedlings grown in a single hole or sponge. The generated annotations were saved in a TXT file comprised of the class associated with each targeted region, and the bbox values were used to draw a rectangular box around the detected region.

### 3.4. Deep Learning Detection System

**Figure 5:**
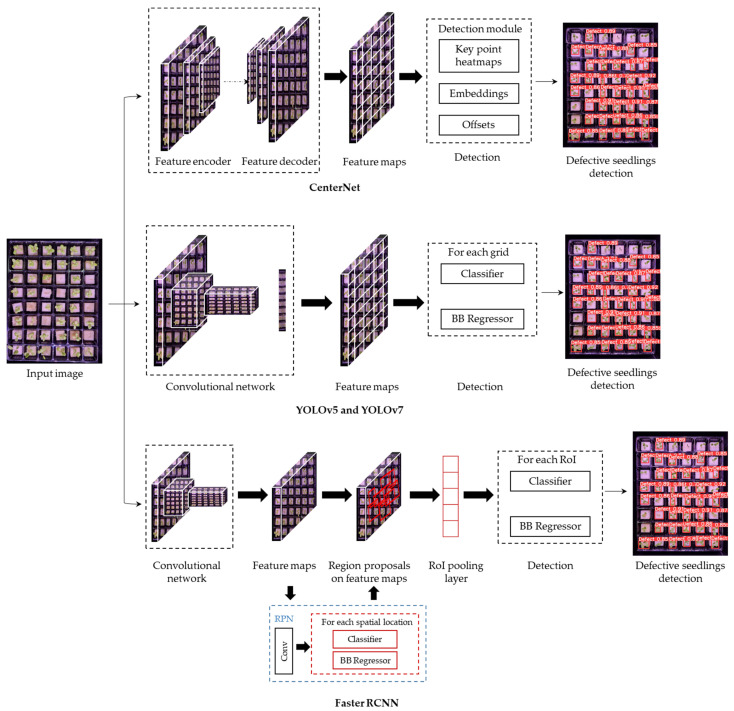
Overall framework for the detection of defective lettuce seedlings using the CenterNet, YOLO, and faster R-CNN models.

In this study, several object-detection models were employed to detect defective lettuce seedlings. The models were CenterNet, YOLOv5, YOLOv7, and faster R-CNN (Figure 5).

#### 3.4.1. CenterNet

**Figure 6:**
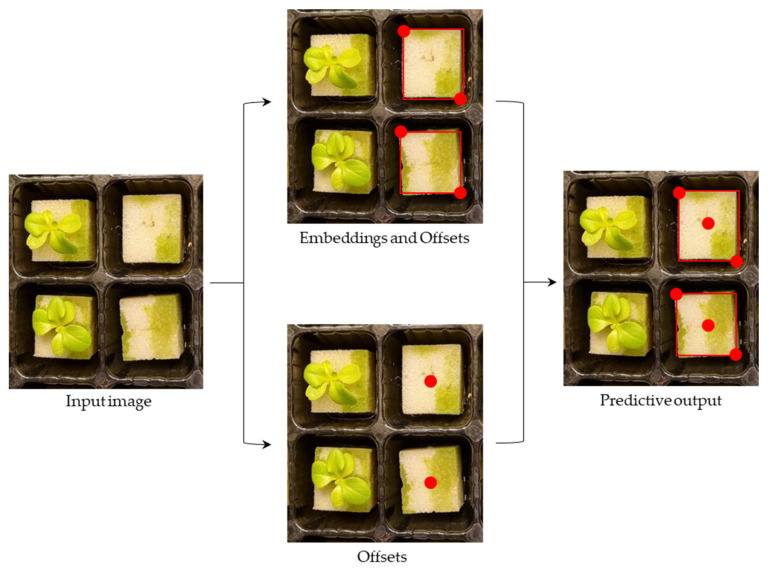
The CenterNet prediction process in detecting defective seedlings based on triplet key points.

CenterNet is an object-detection method that operates in a single stage and does not rely on predefined boxes for object detection. Instead, the CenterNet model uses a heatmap to represent each object as a single point, which corresponds to the center of the object’s bounding box. The defective-seedling-detection process of CenterNet began with the input image, which was first encoded by the CenterNet backbone network and produced a down-sampling feature map. The decoder then up-sampled the output feature map and improved the image resolution, and the detection layer finally detected the targeted class. The output feature map of the detection layer underwent a series of convolution operations that included a key point heatmap, which was used for predicting the object center point, the offset of the center point, and the size of the target object, resulting in the final prediction of the defective seedling area (Figure 6).

#### 3.4.2. YOLOv5 and YOLOv7

YOLOv5 is an improved version of the object-detection algorithm developed by Ultralytics that was built based on its predecessor, YOLOv3. The architecture of YOLOv5 is based on a backbone network that extracts features from an input image, followed by a detection head that makes predictions based on those features. The backbone network used in YOLOv5 is a modified version of the CSPNet architecture, which is designed to improve the efficiency of feature extraction. YOLOv7 is one of the recent models of the YOLO series developed in 2022. It boasts highly improved and accurate object-detection performance by utilizing the extended-ELAN backbone as an innovative approach to improving the network’s self-learning ability while preserving the original gradient path. Additionally, YOLOv7 utilizes a cascade-based model-scaling method to generate models of appropriate scales for specific tasks, thus meeting the detection requirements.

**Figure 7:**
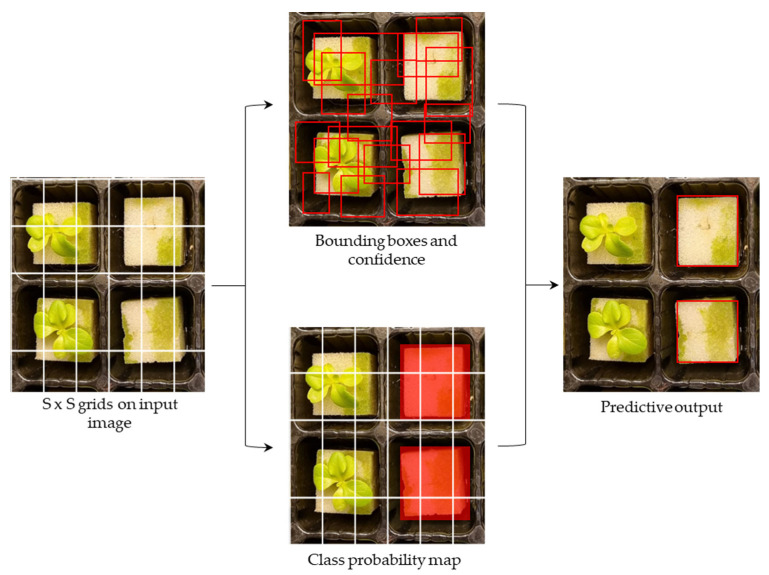
The YOLO prediction process in detecting defective seedlings based on bounding boxes.

In the YOLO model detection processes, defective seedlings were detected by first discretizing the input image of lettuce seedlings into S × S equally spaced grids. Next, the model generated multiple predictive bounding boxes and assigned a confidence score to each box if the defective seedling area fell within the corresponding grid cell. After the network predicted the anchor boxes, non-maximum suppression (NMS) was applied to remove redundant predictions and ensure that the algorithm selected the most accurate prediction that corresponded to the actual location of the defective seedlings. Finally, the remaining predictions were combined to obtain the final set of bounding boxes for the detected defective seedling locations (Figure 7).

#### 3.4.3. Faster R-CNN

**Figure 8:**
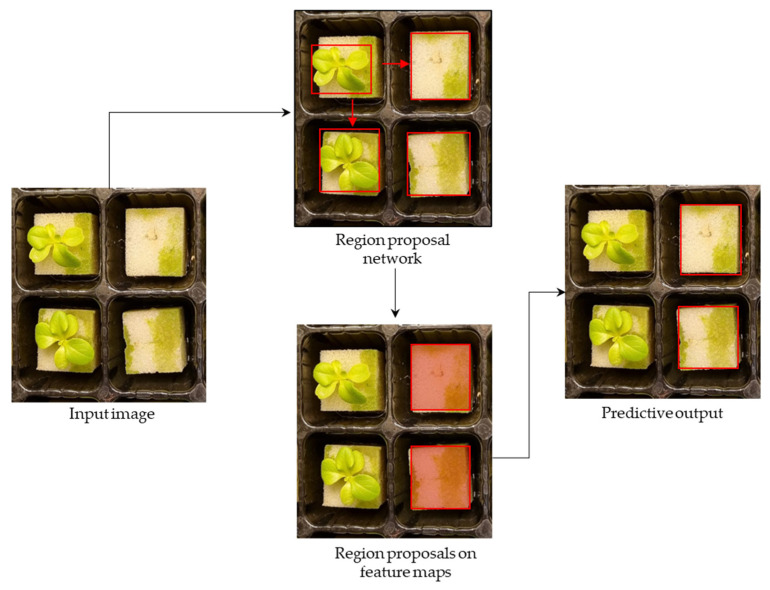
The faster R-CNN prediction process in detecting defective seedlings based on the RPN and fast R-CNN detector.

Faster R-CNN is a two-stage object-detection algorithm that consists of two modules: a region proposal network (RPN) and a fast R-CNN detector. The RPN generates object proposals that are then used as input to the fast R-CNN detector to predict the final object class and bounding box. In predicting defective seedlings, the RPN takes a labeled defective seedling image as input and outputs a set of object proposals by predicting objectness scores and bounding box offsets for a set of anchor boxes at different scales and aspect ratios. These anchor boxes served as the reference boxes of defective seedlings and were used to generate proposals. The proposals generated by the RPN were then used as input to the object-detection network, fast R-CNN, which classified the proposals and refined their bounding box coordinates. The fast R-CNN detector takes an image and a set of proposals as input and outputs the final object class and bounding box for each proposal. The detector model first extracted a fixed-length feature vector from each proposal using a region of interest (RoI) pooling layer. The feature vectors were then fed into a fully connected network that predicted the defective seedling location and bounding box offset (Figure 8).

### 3.5. Model Performance Evaluation

**Figure 9:**
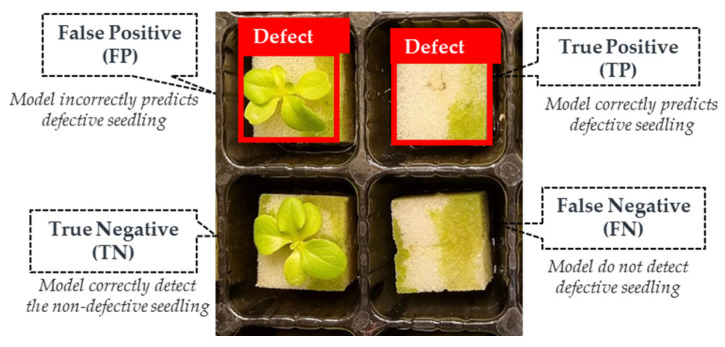
Confusion matrices for detecting defective seedlings.

In this paper, the validation and testing results of object-detection based on the aforementioned deep learning models are presented using a confusion matrix. The confusion matrix consists of four possible outcomes: true positive (TP), false positive (FP), true negative (TN), and false negative (FN). A TP referred to the model accurately identifying defective seedlings, while an FP indicated that the network mistakenly predicted the presence of defective seedlings. In contrast, a TN represented the network correctly identifying images without defective seedlings, while FN referred to the model failing to detect the actual presence of defective seedlings (Figure 9).

To assess the ability of the models to detect defective lettuce seedlings under various indoor farm lighting conditions, precision and recall were computed using the confusion matrix. Precision (P) signifies that the model correctly identified the defective seedlings among all positive and false predictions made by the model, which can be determined using the equation below:

(1) $$\mathrm{Precision}=\frac{\mathrm{TP}}{\mathrm{TP}+\mathrm{FP}}$$

Recall (R) indicates the effectiveness of the model in correctly identifying the defective seedlings among all positive and undetected targets.

(2) $$\mathrm{Recall}=\frac{\mathrm{TP}}{\mathrm{TP}+\mathrm{FN}}$$

Average precision (AP) provides a measure of the model’s performance in terms of precision and recall and is calculated by computing the area under the precision–recall curve and mean average precision (mAP) is the average of APs for all classes.

(3) $$\mathrm{mAP}=\frac{\sum_{1}^{N}\int_{0}^{1}P(R)\mathrm{dR}}{N}$$

where N is the number of classes, and since there is only one class in our dataset, which is defective seedlings, N = 1.

### 3.6. Training Environment Details

The CenterNet and faster R-CNN models were trained using a desktop PC equipped with an NVIDIA^®^ GeForce RTX 2060™ GPU (Nvidia Corporation, Santa Clara, CA, USA), Intel(R) Core (TM) i7-10750H CPU and 32 GB of RAM. The labeled images in TXT format were initially converted into JSON and XML formats for training CenterNet and faster R-CNN, respectively. The YOLOv5 and YOLOv7 models were trained in TXT format input using the cloud-based platform Google Colaboratory, which is a web-integrated development environment (IDE) that leverages the GPU of Tesla P100-PCIE 16 GB.

## 4. Results and Analysis

### 4.1. Model Training and Testing

During the model training, a total of 2205 datasets were used, out of which 2040 images were used for training, and 165 original images were used for validation to adjust the parameters. The evaluation indicators were verified and validated on the original testing set after model weights were obtained. The original images were used as a cross-validation process to avoid duplication of images, which may lead to data leaking and affect the true performance of the model. This research utilized transfer learning by adopting pretrained weights from each tested deep learning model, as shown in Table 1, to establish the detection model of defective seedlings. Transfer learning serves as an effective strategy to expedite the development process and take advantage of the prior knowledge in recognizing general visual features (e.g., colors, edges, and blobs) captured by pre-existing models [41]. This allows for faster convergence during model training. The training settings for all the models were adjusted according to the suitability of the model and dataset, as indicated in Table 1.

#### 4.1.1. Model Training Results

**Figure 10:**
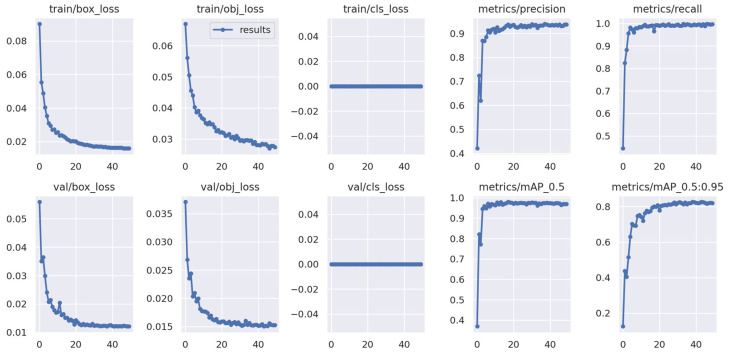
Training performance of datasets using the YOLOv5 algorithm.

**Figure 11:**
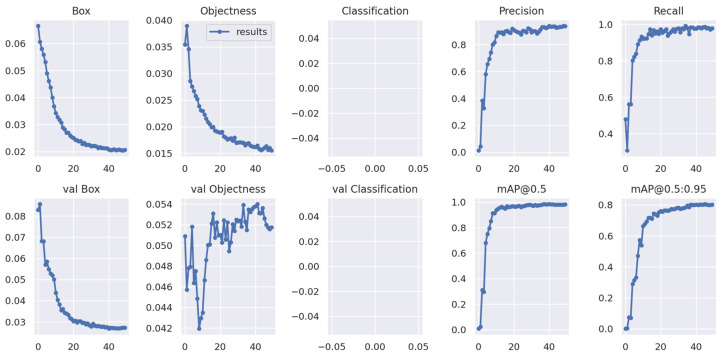
Training performance of datasets using the YOLOv7 algorithm.

Both YOLOv5 and YOLOv7 were trained with 50 epochs. YOLOv5 took two hours to complete the training process and YOLOv7 almost one hour. Overall, both YOLO models showed promising training performances based on loss and mAP. The training curves demonstrated a steady decrease in both losses and an increase in mAP over time, indicating that the algorithms improved their accuracy and became better at detecting defective seedlings in images (Figure 10 and Figure 11).

CenterNet completed the training process within 3 h for 50 epochs, and faster RCNN took five hours to complete the 10,000 iterations. The comparison of validation results at 50% IoU in Table 2 shows that the faster R-CNN model performed slightly better in training than the CenterNet, YOLOv5, and YOLOv7 models. Specifically, the faster R-CNN model had the highest training mAP of 98.2%. All other models, namely CenterNet, YOLOv5, and YOLOv7, had training mAP ranges within 81.2% to 97.3% (Table 2).

#### 4.1.2. Model Testing Results

The performances were compared and evaluated further based on the detection model that was run on 83 unseen and original testing image datasets (Table 3). Based on the testing results, the YOLOv7 model had the highest accuracy with a mAP of 97.2%, followed by YOLOv5 and faster R-CNN with mAPs of 96.5% and 88.6%, respectively. On the other hand, CenterNet had the lowest detection accuracy with a mAP of 82.8%. These results suggest that YOLOv7 outperformed all other tested models in terms of accuracy for detecting defective lettuce seedlings in indoor farming environments.

The sizes of CenterNet, YOLOv5, YOLOv7, and faster R-CNN were 75.1 MB, 10.2 MB, 11.7 MB, and 1.2 GB, respectively (Table 3). These findings provide important insights into selecting the most suitable algorithm for defective seedlings in indoor farms, considering factors such as detection accuracy, model size, and computational requirements for application. This could serve as a reference for developing an efficient automatic sorting system for seedling management in indoor farms.

### 4.2. Detection Results under Different Lighting Conditions

To understand the performance of each model more intuitively, the test dataset was divided into three lighting categories: white fluorescent, red/blue LEDs, and red/blue/white LEDs. There were 39 images under white fluorescent light, 28 images under red/blue LEDs, and 16 under red/blue/white LEDs. Based on the results presented in Table 4, all the tested models showed promising performances in detecting defective lettuce seedlings under different lighting conditions. Specifically, the models exhibited higher precision in their detections under red/blue/white compared to other lighting conditions.

YOLOv7 had the highest accuracy with a mAP of 95.8% in detecting defective seedlings under white light, followed by YOLOv5, faster R-CNN, and CenterNet with mAP values of 94.9%, 86.6%, and 81.7%, respectively. Under red/blue lighting, YOLOv5 performed 0.5% slightly better than YOLOv7. YOLOv7 also had the highest accuracy in detecting defective lettuce seedlings under red/blue/white light conditions with mAP values of 98.8%. Meanwhile, CenterNet provided the lowest accuracy in detecting seedlings under all lighting conditions compared to the other tested models.

**Figure 12:**
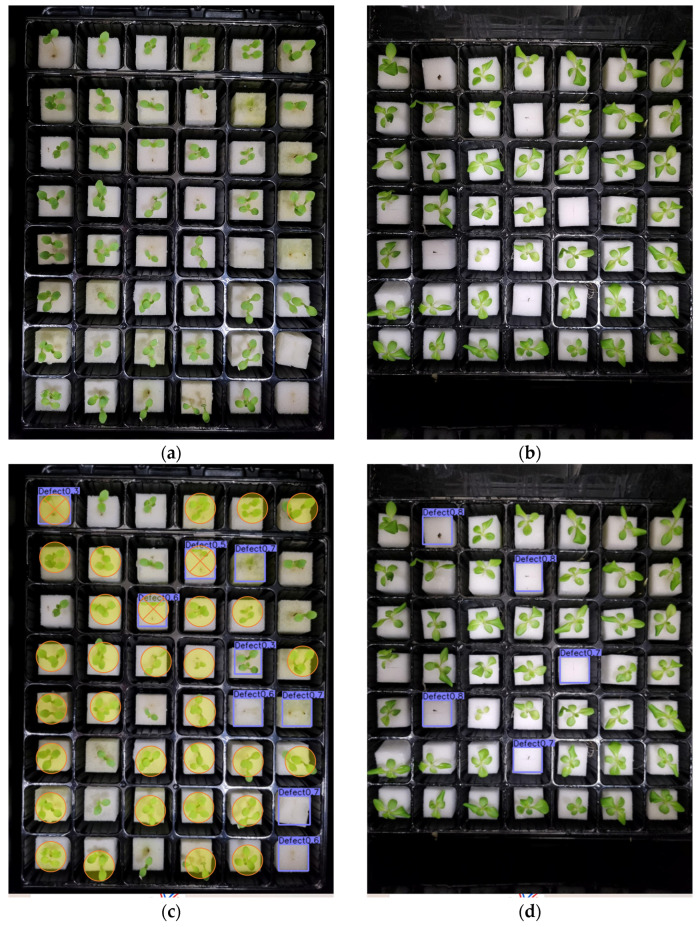

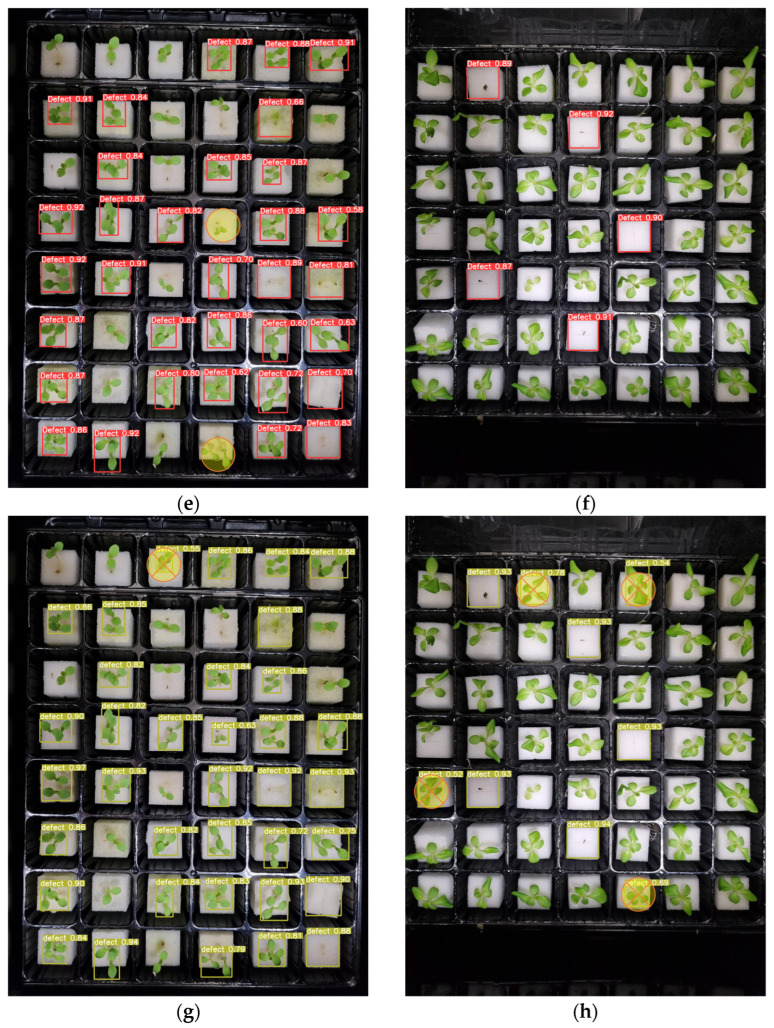

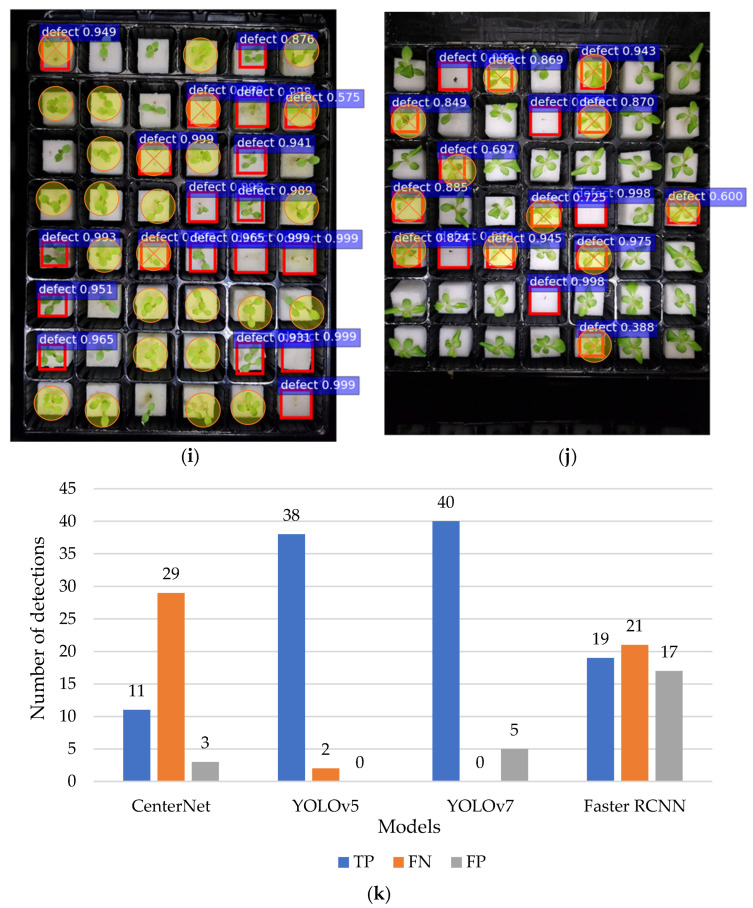
Test examples of the detection of defective lettuce seedlings under white light conditions from (**a**,**b**) original image; (**c**,**d**) CenterNet; (**e**,**f**) YOLOv5; (**g**,**h**) YOLOv7; (**i**,**j**) faster R-CNN; and (**k**) summary of the detection results. (The square mark in the figure refers to detected seedlings, and the circle mark indicates false and misdetected seedlings.)

**Figure 13:**
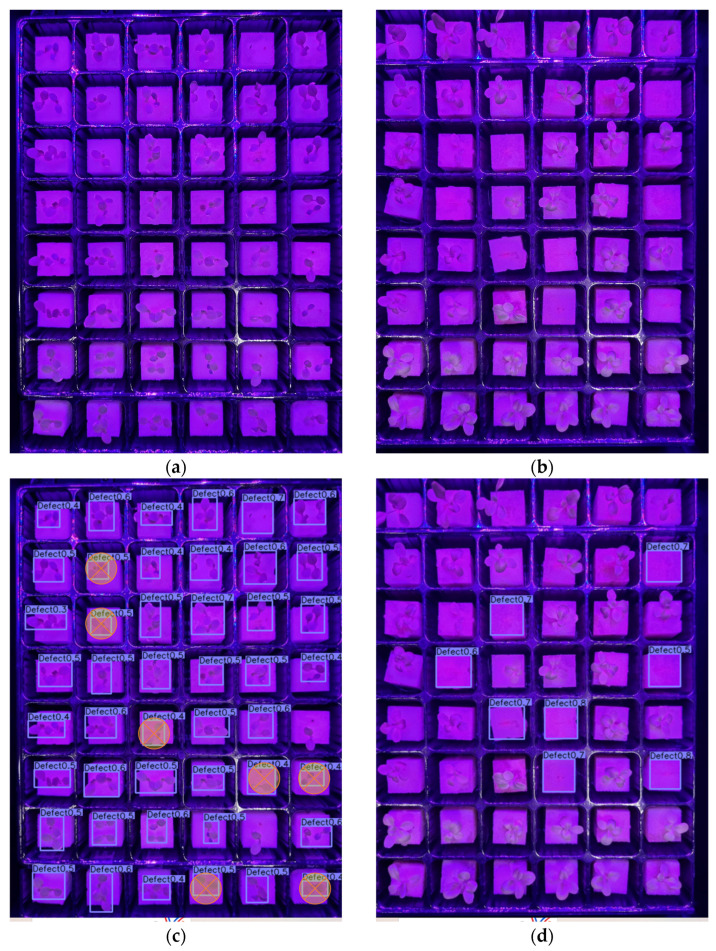

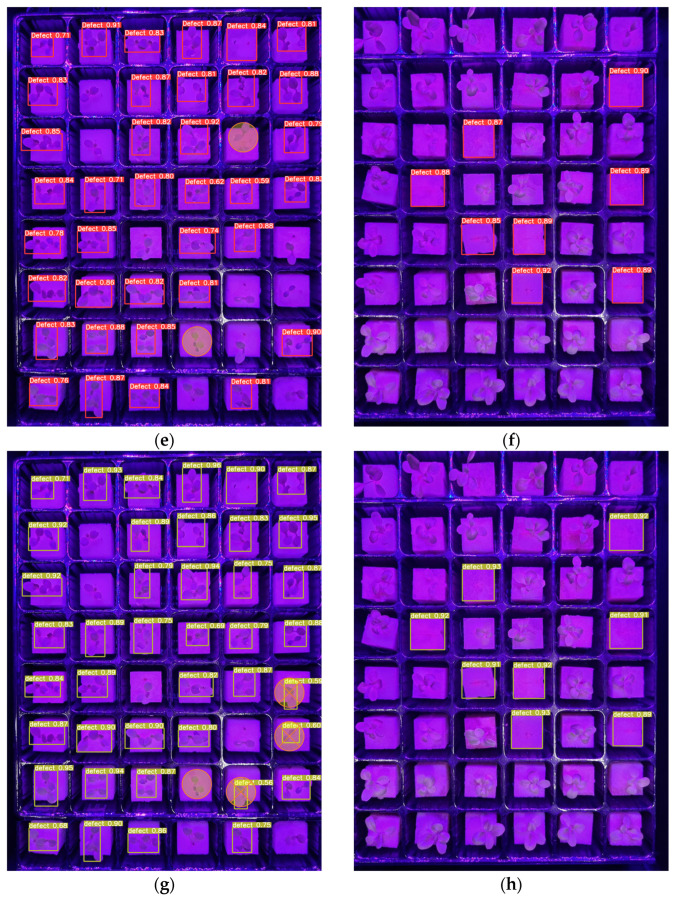

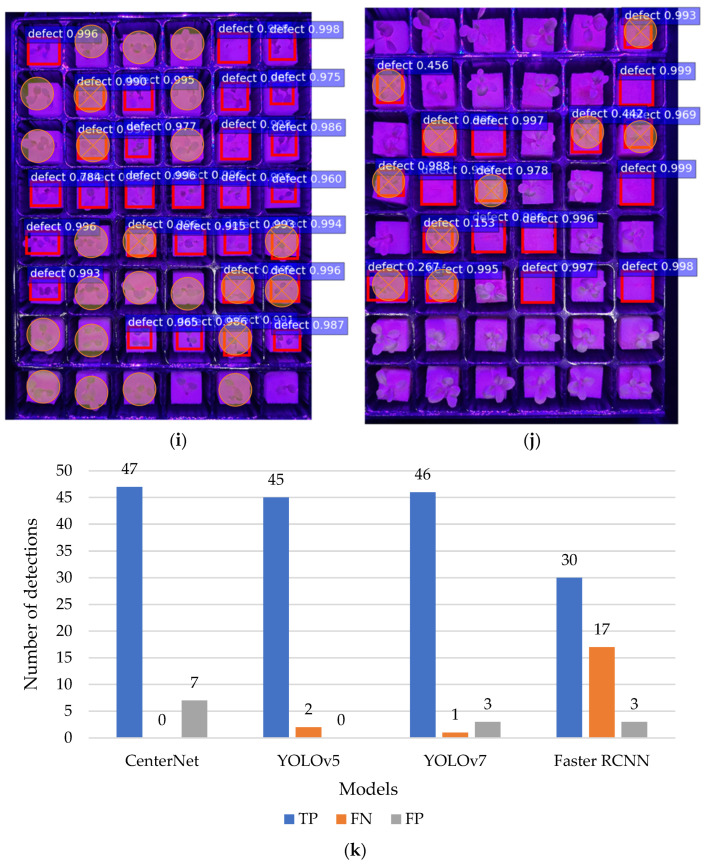
Test examples of the detection of defective lettuce seedlings under red and blue light conditions from (**a**,**b**) original image; (**c**,**d**) CenterNet; (**e**,**f**) YOLOv5; (**g**,**h**) YOLOv7; (**i**,**j**) faster R-CNN; and (**k**) summary of the detection results. (The square mark in the figure refers to detected seedlings, and the circle mark indicates false and misdetected seedlings.)

**Figure 14:**
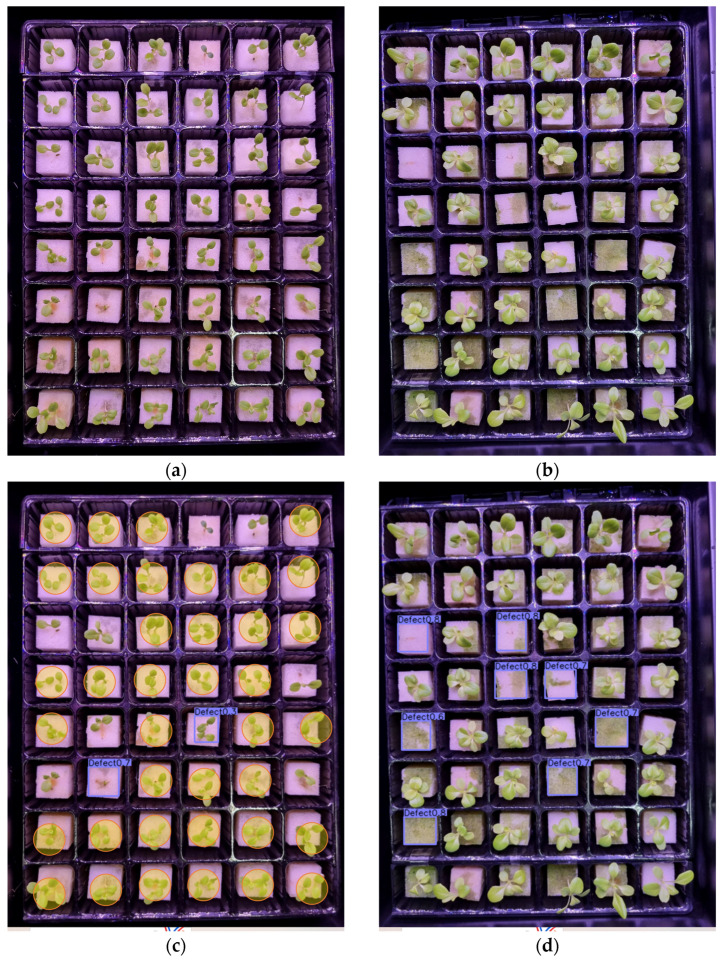

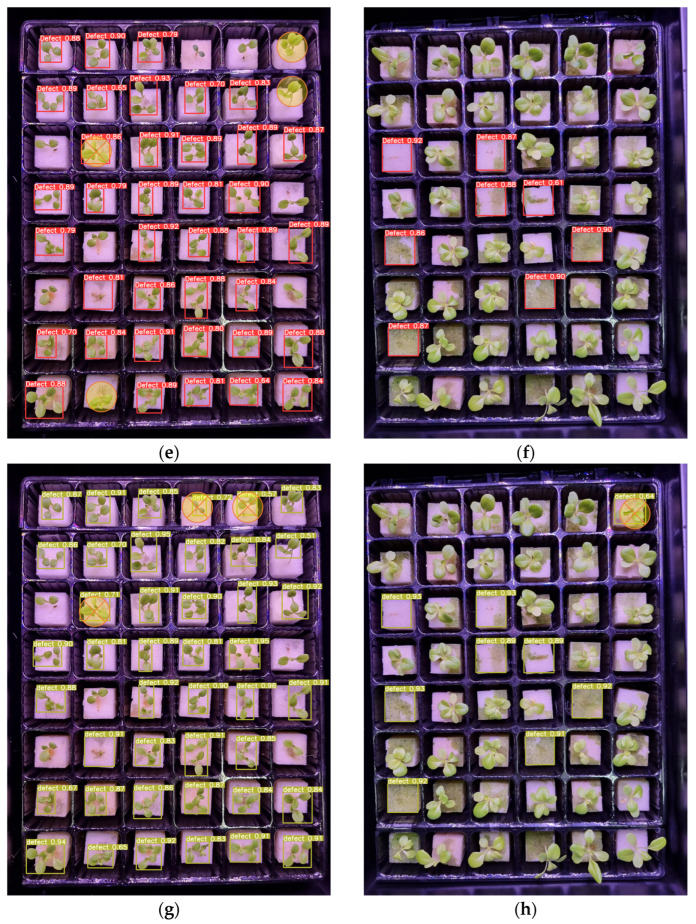

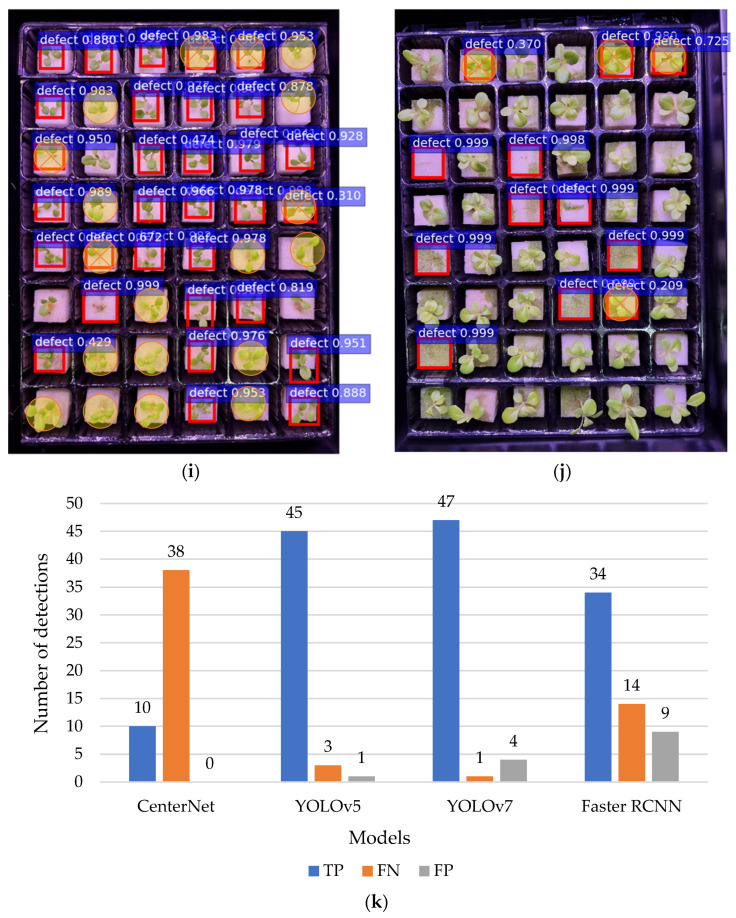
Test examples of the detection of defective lettuce seedlings under red, blue, and white light conditions from (**a**,**b**) original image; (**c**,**d**) CenterNet; (**e**,**f**) YOLOv5; (**g**,**h**) YOLOv7; (**i**,**j**) faster R-CNN; and (**k**) summary of the detection results. (The square mark in the figure refers to detected seedlings, and the circle mark indicates false and misdetected seedlings.)

Figure 12, Figure 13 and Figure 14 present examples of the detection results of the four models on defective lettuce seedling images taken under different light conditions. Out of 40 total defective seedlings under white light, YOLOv7 was able to detect all the defective seedlings, followed by YOLOv5 (38 detections). However, YOLOv7 had five false detections (Figure 12g,h). Both CenterNet (Figure 12c,d) and faster R-CNN (Figure 12i,j) performed the worst, detecting less than half of the total number of defective seedlings.

For red/blue lighting, CenterNet detected all 47 defective seedlings; however, it had seven FPs (Figure 13g,h). YOLOv7 and YOLOv5 had 46 and 45 detections, repectively. The YOLOv7 model’s detection effect was higher than that of YOLOv5 with only one misdetection (Figure 13e,f). Faster R-CNN only detected 30 defective seedlings under red/blue lighting with 17 missing detections and 3 FPs (Figure 13i,j).

Figure 14 illustrates the model detection of defective seedlings under red/blue/white lighting with a total detection of 48. Once again, YOLOv7 exhibited the best detection performance among the models, achieving the highest detection number of 47 (Figure 14g,h). In contrast, both CenterNet (Figure 14c,d) and faster R-CNN (Figure 14i,j) had a misdetection rate of over 50%, indicating poor performance in accurately identifying the defective seedlings under red/blue/white lighting conditions.

## 5. Discussion

### 5.1. Analysis and Comparison of Different Target Detection Algorithms

Manually identifying defective seedlings for mass production of indoor farming can be a challenging task, especially in complex scenes of lighting conditions that are not suitable for a human to work under for a long time. In this work, defective seedlings grown in indoor environment were detected using several SOTA deep-learning-based object models. The models used in the study were CenterNet, YOLOv5, YOLOv7, and faster R-CNN. The YOLOv7 model outperformed the other three detection algorithms in terms of detection accuracy, as evidenced by its highest mAP of 97.2%. Regarding model size, the faster R-CNN model was the largest model among the other object-detection models. This is because the faster R-CNN model used a two-stage detector that involved an additional step of region proposal generation in the input image of defective seedlings before predicting the defective seedling class and bounding boxes. This added step made the model more complex, which led to a larger model size. The number of model parameters is a crucial factor to consider when deploying a model in real-world scenarios. As the number of parameters increased, more RAM was required to store and run the model. Although high-capacity memory devices are now accessible, their high cost and compatibility difficulties may pose limitations to practical applications. Therefore, for defective seedling applications, it is more practical to use lightweight models with lower memory requirements and higher accuracy. Overall, after comparing the detection performance of all four detection models, the YOLOv7 model can be considered for convenient deployment on embedded devices for real-time automated sorting applications in identifying defective seedlings in indoor farm environments.

### 5.2. Analysis of the Influence of Different Indoor Lighting Conditions on the Detection Results

Different spectra and intensities are important for plant growth in indoor farming because different stages of plant growth require varying amounts and types of light. Plants undergo different physiological processes at different growth stages, and providing the appropriate light spectra and intensities can optimize their growth, development, and productivity [42,43]. For instance, during the early stages of plant growth, such as germination and seedling establishment, plants require higher levels of blue light to promote healthy root and shoot development. The blue light spectrum helps regulate photomorphogenic responses, promoting compact and vigorous seedlings [9]. However, the utilization of red and blue LEDs in plant lighting systems typically results in plants displaying a purplish-grey color to the human eye. This problem poses a challenge when visually assessing the health of these plants, including the identification of disease symptoms, nutritional deficiencies, and physiological disorders [9]. Hence, it is important to have a seedling-detection system that can perform under complex lighting in indoor farming and ensure seedling quality assessment for effective management in indoor farming.

It is worth noting that different lighting conditions can have a significant impact on the performance of object-detection models, as they can affect the contrast, color, and texture of the objects in the image. The detection effect under red/blue/white was better than the detection effect under white. This is because the red/blue/white lighting conditions provide better illumination and contrast, allowing the models to more easily detect the plants’ features accurately. On the other hand, defective seedling detection is a challenging task under red and blue lighting conditions due to the complexity of the scenes and the difficulty in differentiating the color of the targeted seedlings from the background, which results in a lower detection effect under red and blue lighting. The limited spectrum of light in red and blue LED lighting conditions can cause color distortion and may also affect the plant appearance, making it more difficult for both humans and models to detect defective seedlings accurately. In addition to these challenges, there may also be errors and inconsistencies during the labeling process, which can affect model accuracy. For example, some defective seedling locations, especially under red and blue light conditions, may not be labeled correctly, leading to false negatives or positives. It is crucial to ensure labeling process accuracy by implementing quality-control measures and minimizing errors and inconsistencies to address these challenges.

Furthermore, most of the missed detections of defective seedlings occurred while the defective seedling situation was double or multiple. This is because the occlusion of leaves in double or multiple seedlings may be very similar to that of the individual seedling at a later stage of growth, which can cause inaccuracies when deep learning algorithms identify them as defective. To address this challenge, it may be helpful to take the image from a different angle, not only from the top but also from the side view, to enhance the learning features of the model.

### 5.3. Study Limitations and Recommendations for Improvement

There are still some limitations to this study that should not be overlooked. The training dataset was found to have an imbalance in terms of the number of images of seedlings captured under different light conditions, with the lowest number captured under white lights. This imbalance can cause the model to not extract sufficient features for some targets during the training process, which ultimately may affect the model’s detection performance, resulting in lower detection accuracy under white light conditions. To improve accuracy, we suggest balancing the dataset by including more images captured under white light conditions. This issue can also be addressed by using techniques such as oversampling or undersampling to balance the dataset [44].

The next work will consider identifying different types of defective seedlings by classifying them into separate categories, including multiple seedlings and dead or non-germinating seeds. This classification can assist in the thinning process for multiple seedlings, potentially increasing seedling survival rates and saving more seedlings. Meanwhile, in the case of dead or non-germinating seeds, they can be easily discarded. These insights can then be used to refine and improve the performance of deep learning models in detecting defective seedlings under different indoor lighting conditions, paving the way for the development of more accurate and reliable seedling-detection systems for indoor farming.

In future research, to realize automated seedling sorting for real-time application in indoor farm environments, a defect-detection system can be incorporated with multiple object tracking (MOT) algorithms. The MOT can be used to assign a unique ID for each detected object [45]. Multiple hypothesis tracking (MHT) [46], kernelized correlation filter (KCF) tracker [47], and deep learning simple online and real-time tracking (deep SORT) [48] are some examples of MOT algorithms that can be considered together with deep learning object detectors to track seedlings. This combined method can be implemented to develop an automatic platform, such as an end-effector for a seedling-sorting machine, which can replace the tedious manual seedling-sorting task. Incorporating both detection and MOT algorithms can help the end-effector to track and sort individual seedlings accurately. Developing such automatic systems create opportunity in advanced-precision agriculture systems and ultimately improve indoor seedling-management efficiency.

## 6. Conclusions

Manual sorting of defective seedlings is a tedious task for indoor lettuce production under different lighting conditions. In this regard, a detection model for defective lettuce seedlings grown in indoor environments was developed for the application of an automatic sorting system. An indoor system was built for germinating seedlings under three different indoor lighting conditions: red/blue LEDs, red/blue/white LEDs, and white fluorescent. Four types of SOTA deep learning models based on a one-stage detector, namely, CenterNet, YOLOv5, and YOLOv7, and a two-stage detector, faster RCNN, were used and evaluated for the automatic detection of defective lettuce seedlings. The following conclusions were drawn from this research:

1. Defective seedlings under a different range of indoor lighting conditions were detected using deep learning algorithms;
2. The detection accuracies of CenterNet, YOLOv5, YOLOv7, and faster RCNN was 82.8%, 96.5%, 97.2%, and 88.6%, respectively;
3. In terms of detection under white lights, YOLOv7 had the highest accuracy of 95.8%. YOLOv5 had the highest accuracy (99.0%) under red/blue lights. YOLOv7 also achieved maximum accuracy of 98.8% under red/blue/white lights;
4. The YOLOv7 model had the best overall results, with an mAP of 97.2%, and has the potential to be employed for automated sorting systems.

The effectiveness of the defective-lettuce-seedling-detection system can be further enhanced by integration with MOT algorithms to develop an end-effector for a smart seedling-sorting machine. In this way, the system can operate autonomously, reducing the need for manual labor and increasing the overall precision and efficiency of indoor seedling management.

## Figures and Tables

**Table 1:** Parameter setup during the training process.

Model		Input Size	Epoch	Batch
CenterNet	ctdet_pascal_dla_512	512 × 512	50	-
YOLOv5	CSP-Darknet53	512 × 512	50	-
YOLOv7	Extended-ELAN	512 × 512	50	-
Faster R-CNN	VGG-16	512 × 512	-	10,000

**Table 2:** CenterNet, YOLOv5, YOLOv7, and faster RCNN training model evaluation for the detection of defective seedlings.

Model	Precision	Recall	mAP @0.5
CenterNet	-	0.850	0.812
YOLOv5	0.936	0.990	0.973
YOLOv7	0.937	0.989	0.966
Faster R-CNN	0.853	1.000	0.982

**Table 3:** CenterNet, YOLOv5, YOLOv7, and faster RCNN model evaluation for detecting defective seedlings on the testing dataset.

Model	Precision	Recall	mAP @0.5	Model Size
CenterNet	-	0.880	0.828	75.1 MB
YOLOv5	0.936	0.980	0.965	10.2 MB
YOLOv7	0.939	0.965	0.972	11.7 MB
Faster R-CNN	0.850	0.996	0.886	1.2 GB

**Table 4:** The mAP of the defective lettuce seedlings under different indoor lighting conditions.

Model	White	Red/Blue	Red/Blue/White
CenterNet	0.817	0.826	0.883
YOLOv5	0.949	0.990	0.980
YOLOv7	0.958	0.985	0.988
Faster R-CNN	0.866	0.981	0.912

## Data Availability

The dataset that was generated and analyzed during this study is available from the corresponding author upon reasonable request, but restrictions apply to the data reproducibility and commercially confident details.

## Abbreviations

The following abbreviations are used in this manuscript:

AI	Artificial intelligence
CNN	Convolutional neural network
DL	Deep learning
SOTA	State-of-the-art
SSD	Single-shot multibox detector
YOLO	You only look once
NMS	Non-maximum suppression
RPN	Region proposal network
RoI	Region of interest
TP	True positive
TN	True negative
FP	False positive
FN	False negative
MOT	Multiple object tracking
MHT	Multiple hypothesis tracking
KCF	Kernelized correlation filter
SORT	Simple online and real-time tracking
